# The unfavorable role of titanium particles released from dental implants

**DOI:** 10.7150/ntno.56401

**Published:** 2021-03-10

**Authors:** Zilan Zhou, Quan Shi, Jie Wang, Xiaohang Chen, Yujia Hao, Yuan Zhang, Xing Wang

**Affiliations:** 1 Shanxi Medical University School and Hospital of Stomatology, Taiyuan 030001, China.; 2 Shanxi Province Key Laboratory of Oral Diseases Prevention and New Materials, Taiyuan 030001, China.; 3 Institute of Stomatology, First Medical Center, Chinese PLA General Hospital, Beijing, China.

**Keywords:** Titanium Particles, Chemical Corrosion, Surface Wear, Surface Modification.

## Abstract

Titanium is considered to be a metal material with the best biological safety. Studies have proved that the titanium implanted in the bone continuously releases titanium particles (Ti particles), significantly increasing the total titanium content in human body. Generally, Ti particles are released slowly without causing a systemic immune response. However, the continuous increased local concentration may result in damage to the intraepithelial homeostasis, aggravation of inflammatory reaction in the surrounding tissues, bone resorption and implant detachment. They also migrate with blood flow and aggregate in the distal organ. The release of Ti particles is affected by the score of the implant surface structure, microenvironment wear and corrosion, medical operation wear, and so on, but the specific mechanism is not clear. Thus, it difficult to prevent the release completely. This paper reviews the causes of the Ti particles formation, the damage to the surrounding tissue, and its mechanism, in particular, methods for reducing the release and toxicity of the Ti particles.

## Introduction

Dental caries, trauma, and other tooth loss causes seriously affect the quality of life of patients. In the mid-1950s, Branemark and Albrektsson first used high-purity titanium as an implant material and put forward the classical osseointegration theory (titanium direct contact with bone tissue without fibrous tissue intervention) 1. Nowadays, titanium implants have been utilized in a higher frequency to replace missing teeth, because it provides a long-term masticatory function and an excellent aesthetic effect 2. The number of dental patients increases by 5 million each year worldwide 3. Moreover, titanium is currently considered the first-rate metal material with biological safety, and almost 1,000 tons of titanium have been applied clinically in different forms, such as artificial joints, cochlear implants, and heart valves, every year 4.

In a healthy human body, titanium content should not exceed 15 mg per 70 kg body weight. Studied have demonstrated that titanium implants in bones could continuously release titanium particles (Ti particles) 5, 6, which may last from a few hours to several months. Generally, these particles are highly insoluble and difficult to eliminate from the body, usually distributed in the hard and soft tissue around the implant 7. The release of Ti particles is often neglected because the release rate is relatively slow without induction of a systemic immune response 8. Once the concentration of local Ti particles is excessive, it will destroy oral intraepithelial homeostasis, aggravate inflammation in surrounding tissues, and lead to a dynamic imbalance of osteoblasts and osteoclasts 9, 10. Furthermore, the released Ti particles are not confined in the tissue around the implant, even migrate with the blood, and gradually accumulate in the distal organs 6, 11-13, causing systemic allergies and allergic reactions.

At present, various techniques have been used to increase surface roughness and hydrophilicity, such as sandblasting acid etching (SLA), plasma spraying, electrophoretic deposition, and other methods 4. However, none of them could thoroughly prevent the release of Ti particles from the implant. The influencing factors of the release are related to the surface structure of the implant, micro-environment wear and corrosion, medical surgery wear and so on. This paper reviews the cause of Ti particles released from dental implants, the mechanisms and the damage to the surrounding cells and tissues, as well as methods to reduce the release and toxicity of Ti particles.

## 1. Ti particles

### 1.1. Definition

Titanium is generally accepted to be the preferred metal material in dental implants. However, once exposed to air, the titanium implant surface spontaneously forms a stable titanium oxide layer. The corrosion resistance of implant comes from the oxide layer that protects the implant from the surrounding tissue. Various factors destroy the titanium oxide layer 14, releasing Ti particles from the surface. Those particles are not entirely bioinert material, and are distributed in cells and tissues. It has been reported that the presence of Ti particles may be harmful and trigger a series of biological reactions (Table 1, Table 2) 15-17.

Ti particles have multiple shapes, such as round and slender 2, with sizes of 15 nm to 45 μm. Nanoparticles and micron Ti particles are more common than millimeter particles 18, 19. It has been reported that the size of Ti particles decreases with the increase of distance from the titanium implant 20, 21.

### 1.2. Distribution

**Figure 1:**
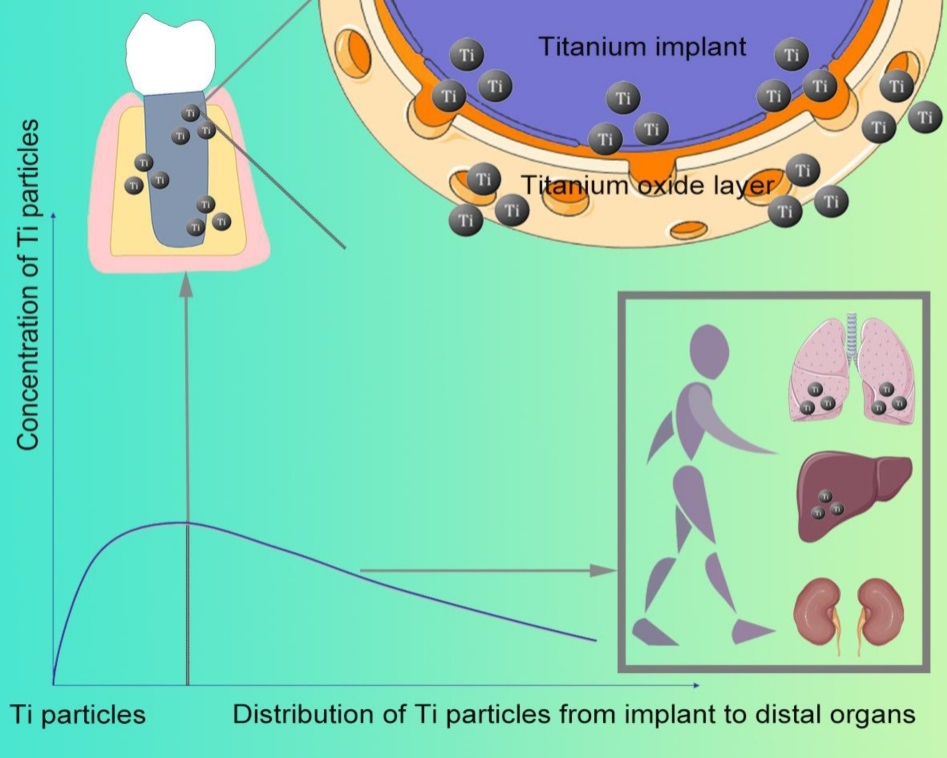
The relationship between Ti particle distribution and concentration

After implant placement, Ti particles can be detected on the bone surface and soft tissues around the jaw implant even a few hours. The concentration of Ti particles distributed in the human body is related to the implant distance (Figure 1)-the closer to the implant, the higher the concentration 7. Compared with implants without inflammation, a higher concentration of Ti particles can be detected at the inflammation site of implant 2. Moreover, released from the implant, they enter the blood and migrate to multiple organs throughout the body, which can be found in the submandibular and cervical lymph nodes 6, as well as lung, kidney, and liver (Figure 1).

### 1.3. Detection methods

Exfoliative cytology is a simple, non-invasive, well-tolerated diagnostic technique 22. Cell samples are collected by rotating a microbrush over the surface of the mucosa, and the concentration of Ti particles is measured microchemically using an inductively coupled plasma-mass spectrophotometry (ICP-MS) 23, 24. In addition to detect the Ti particles released from the implant surface, oral exfoliation cytology is a tool for detecting metal particles in the cells that shed mucosa around implants and monitoring dental implant corrosion 25. Laser ablation inductively coupled plasma mass spectroscopy (LA-ICP-MS) is a new technique for determining elements in titanium implants and enable to quantitatively analyze tissue blocks or thin slices placed on different bases, showing the content of resultant two-dimensional map and distribution of elements in the sample. Sajnóg et al. used the LA-ICP-MS to examine the oral mucosa samples around the titanium implants, and revealed that high content of Ti particles is derived from implants 26. Swiatkowska et al. suggested to use the concentration of Ti particles in the blood as a biomarker for implant wear with high-resolution instruments 27. Various analytical methods have been used in labs, but the indication of implant health still remains controversial.

## 2. Cause of release

**Figure 2:**
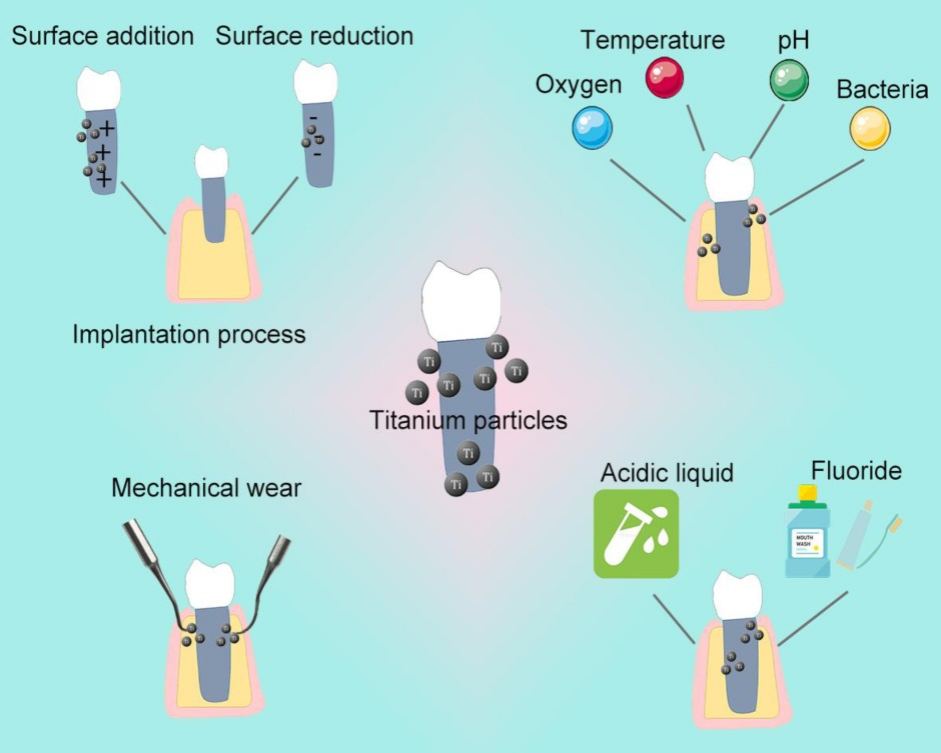
Grounds for the release of Ti particles

Chemical corrosion and Surface wear are two main reasons for the release of Ti particles from implants (Figure 2) 28, 31.

### 2.1. Chemical corrosion

Chemical corrosion is inevitable for almost all the metal implants currently. Williams et al. found that all metals, whether precious or passivated, slowly release metal ions and particles from the surface after long implantation into the human body 32. Sarmiento-González et al. first reported that in the absence of wear and tear 33, partial surface damage of titanium oxide film occurred after implantation for 12 months or longer 34. The content of Ti particles increases in the blood, leading to accumulation in organs. The release in the surrounding tissues is attributed to different temperatures of the oral environment, saliva pH. Moreover, bacterial circulation erodes the titanium oxide layer of implant during long-term use 35.

Saliva acting as a weak electrolyte, simulates an electrochemical cell in the oral cavity, forms a potential difference, and promotes dissolution of the titanium oxide layer. Hjalmarsson et al. reported that the implant surface becomes rough after contact with saliva 36. Further electrochemical corrosion of titanium and its alloys causes crevice corrosion, and finally releases of Ti particles 37, 38. Infections, drugs, food, periodontitis, smoking, and systemic diseases can reduce the normal saliva pH of 6.3-7.0 to <6.0 39, 40. Acidic environment destroys the surface oxidation layer on the implant film, erodes the surface, and finally lead to releases Ti particles even without wear 41-43. Gil et al. observed the microstructure of different dental materials before and after the corrosion process, and found that mechanical load significantly reduces the corrosion resistance of titanium 44. The metal difference between the implant and the superstructure also produces a potential difference, causing gaps, pitting, electrical corrosion, and finally, releasing of Ti particles from implant 45.

Chemical methods reduce bacterial adhesion and eliminate bacterial toxins or byproducts on the implant surface during routine maintenance of implant. However, some daily used drugs have been proved to corrode the surface, causing release of Ti particles. Wheelis et al. found that various oral commonly used drugs, such as citric acid, tetracycline, sodium fluoride can cause different degrees of corrosion on the implant surface 46. Acidic solution (pH <3) or high fluoride concentration (>0.2%) of the above drug mentioned can destroy the oxide film on the surface in a short time, and the Ti particles released 47-53. Moreover, fluoride, often added in toothpastes and gels, reduces the corrosion resistance of metal implants 54, destroy the titanium oxide layer, and promotes its dissolution in the oral electrolysis environment 47.

### 2.2. Surface wear

Friction between the bone tissue and the implant generates mechanical retention force during the implantation process, manifesting as microfracture and compression on the bone tissue side 55. Stress concentration on the implant surface destroys the titanium oxide layer on the body of implant and wares the cover, then releases the Ti particles. Almost all implant implantation processes involve the release associated with implant torque, implant surface roughness 5, surface topography 56, and titanium oxide layer density.

Clinicians use physical instruments, chemical reagents, and lasers to periodically remove proliferating bacteria and their harmful products from the contaminated implant surface 57. Sirinirund et al. found that metal instruments for cleaning the titanium surface can change the finished morphology of titanium surface and induce Ti particles to fall off the surface 58. Metal instruments such as metal curettes and scalers do irreversible damage to the implant 59-64, which may change its original surface morphology, result in chemical changes, and release Ti particles. Augthun et al. also found that after using a curette for 60 seconds, the original surface of implant thread edge became rough 63.

Eger et al. studied the relationship between ultrasonic cleaning and release of Ti particles 3. They found that ultrasonic cleaning of titanium implants causes the release and might also exacerbate peri-implant inflammation. The surface roughness of the implant is changed, influencing formation of biofilm, as well as cells reattachment, which changes the integration process of implant and bone 60. Non-metallic instruments cause minimal changes and damage to the titanium surface, but they are still related to the release of Ti particles 59. Hallmon et al. and Homiak et al. found that it also changes the surface after using the plastic curette many times 61, 62.

## 3. Damage mechanism on cells

### 3.1. Inhibition of cell activity

**Figure 3:**
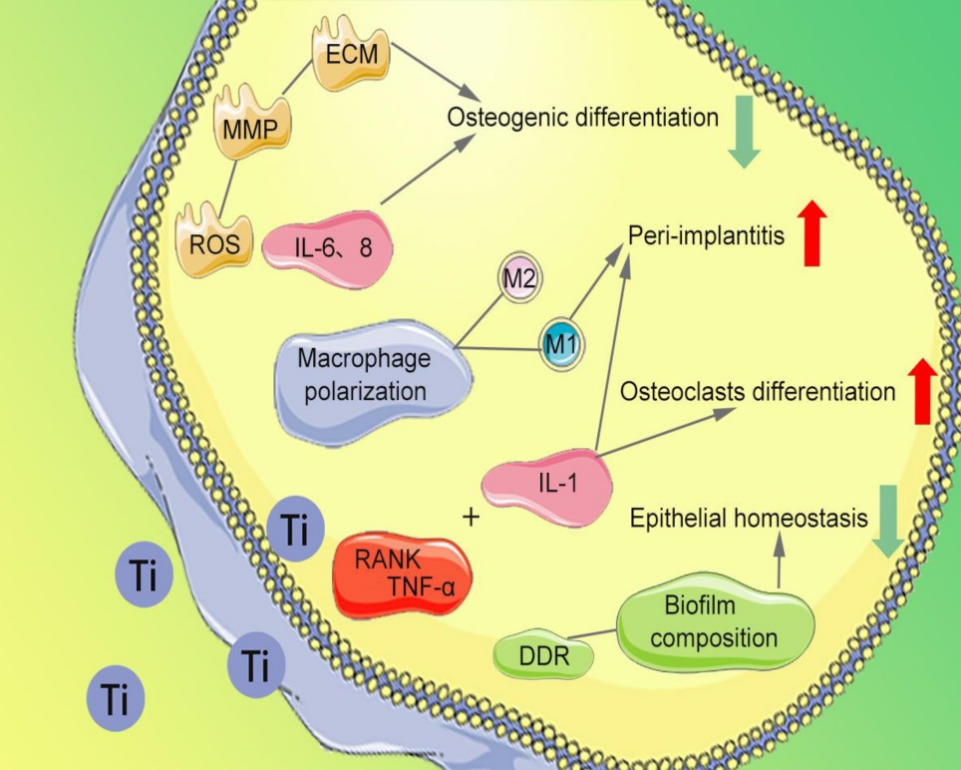
Damage mechanism of Ti particles

The migration, proliferation, and osteogenesis differentiation of bone marrow mesenchymal stem cells (BMSCs) and osteoblasts on the implant surface are the importance guarantee for implant osseointegration 65. Several studies have reported that Ti particles affect the normal cytoskeleton of BMSCs, provoking high levels of reactive oxygen species (ROS) expression, abnormal recruitment of neutrophils 66,67, and production of high levels of matrix metallopeptidase (MMP). The degradation of extracellular matrix (ECM) and inhibition of osteogenesis differentiation of BMSCs are observed. The release of Ti particles promotes increased concentrations of interleukin (IL)-6 and IL-8, thereby inhibiting osteoblast function 68, disrupting the bone balance 47, and subsequently leading to bone resorption (Figure 3). After being co-cultured with BMSCs and human osteoblasts respectively 69, the survival rate and vitality of BMSCs and osteoblasts are decrease. In addition, Happe et al. also found that the activity of osteoblasts is negatively correlated with the concentration of Ti particles 70.

### 3.2. Stimulation of osteoclast differentiation

Severe bone resorption at the implant-bone interface is the most common factor for implant failure. The abnormal aggregation of osteoclast precursor cells is considered to be an essential cause of bone resorption. Ti particles have been proved to inhibit the differentiation of osteoblast precursor cells and promote the bone resorption function of osteoclasts by inducing the differentiation of osteoclasts 71. Wang et al. found that nano-sized Ti particles can inhibit periodontal ligament cells and the osteogenic differentiation of alveolar bone cells 72. It promotes secretion of tumor necrosis factor-alpha (TNF-α), IL-1, and receptor activator of nuclear factor-κB ligand (RANKL), which ultimately promotes differentiation of monocytes into osteoclasts (Figure 3). Furthermore, Ihn et al. found that Ti particles are taken up by osteoclasts with little effect on the activity of osteoclasts 73. However, the bone resorption area of titanium-containing medium group' is significantly more extensive than that of the medium without Ti particles 74. The number of osteoclasts increased with Ti particles concentration between 1×10^4^ and 4.2×10^4^ particles/cm^2^ 73.

### 3.3. Stimulation of macrophages

Ti particles promote the release of inflammatory factors in the tissues around the implant, leading to infiltration of inflammatory cells and triggering a series of immune-inflammatory responses 10, 19, 75. Neutrophils and macrophages take up Ti particles of about 2μm, resulting in recruitment of inflammatory cells to surrounding tissues 76. Pajarinen et al. found that the induced degree of inflammation in surrounding tissues depends on polarization of macrophages 75. Ti particles greatly enhance the overall chemotaxis and inflammatory response of classically activated macrophages (M1) that promote inflammation, while alternatively activated macrophages (M2) that promote regenerative repair are inhibited (Figure 3). Tsutsui et al. found that Ti particles activate macrophages, promote the release of TNF-α and RANKL receptor activators 77, thereby reducing the formation of osteoprotective proteins. Thus, a microenvironment favorable for osteoclast formation and osteolysis is formed, which lead to bone resorption and implant loosening 78.

## 4. Damage mechanism on tissues

### 4.1. Boost peri-implantitis

Peri-implantitis is one of the primary reasons for implant failure and a common complication of dental implant treatment. It can affect the tissues around the dental implants and cause lose bone. Some reports have reported that Ti particles are related to peri-implantitis and can promote inflammation reaction 79, 80. The content of Ti particles in the tissues around the implants is also higher than in other areas 81. Lappas found that metal nanoparticles can induce the abnormal activation of macrophages by regulating host immunity 82, thereby aggravating DNA damage and oxidative stress. Interestingly, some studies have found that metal complex 83, nanoparticles 84 (self-assembly of molecules into nanoparticles, including platinum, aurum, manganese and cerium), and nanoparticles loading drug 23, 24, promote drug internalization, as well as tumor cell necrosis and apoptosis. However, at present, only the negative effect of nanoparticles released from implants has been studied. Ti particles with pro-inflammatory and cytotoxic effects exacerbate the inflammatory response of the tissue surrounding the implant 85, 86, induce peri-implantitis, and destroy the bone homeostasis around the implant, leading to increased bone resorption 3.

### 4.2. Damage to oral epithelial homeostasis

Souza et al. found that Ti particles affect the composition of biofilms (Figure 3) and change the design of microbes, increasing bacteria types 87, including *Streptococcus anginosus*, *Prevotella nigrescens*, *Capnocytophaga sputigena*, *Actinomyces israelli*. Suárez-López et al. found that during implantation, Ti particles released from the surface of the implant and activated the DNA damage response of oral epithelial cells via the DNA damage response (DDR) pathway 88, thereby breaking the barrier of oral epithelial and accumulating bacteria around the implant 89. Bacteria trigger an inflammatory response that further accelerates implant corrosion and the release of Ti particles. The synergistic effect between mechanical force and bacterial biofilm attenuates the titanium oxide layer and damages the surface of implant, thus intensifying the release 90.

## 5. Reduction in the release

### 5.1. Surface modification

Implant surface modification forming a surface that provides rapid bone healing and immediate implant loading is also a research hotspot in implant surface processing techniques that are related to release rate of Ti particles. To promote the osseointegration of the implant and the surrounding bone, the modification methods usually are categorized into surface addition, surface reduction, surface bombardment, and surface oxidation 91.

Deppe et al. found that the surface treated by surface reduction method has less wear than that of the surface addition one 92. Titanium implants with different surfaces are implanted into the femur and tibia of sheep, including smooth titanium (STi), titanium plasma-sprayed (TPS), alumina oxide sandblasted and acid-etched (Al-SLA), zirconium oxide sandblasted, and acid-etched (Zr-SLA). Franchi et al. found that Ti particles detached from the implant surface are visible at the TPS implant-bone interface at 0 and 14 days after implantation 93. Similarly, Weingart et al. also detected Ti particles on the surface of TPS implants implanted in beagles nine months postoperatively 6. Schliephake et al. observed the shedding of Ti particles after implanting STi implants 94, but the release of Ti particles is less than TPS implant. Less Ti particles are released by surface reduction method because the released Ti particles are looser and the damaged titanium oxide layer is easier to reconstruct.

Several reports indicate that the "plus" Ti surface used for deposition of particles (such as TPS) is more abrasion than the "minus" Ti surface (such as STi), which make it easier to release the Ti particles from the implant.

### 5.2. Selection of materials

Titanium implants are mainly used in dental implants, but the superstructure such as abutment has multiple options, including vanadium, aluminum, cobalt, chromium, molybdenum, zirconia. It is reported that materials with different mechanical properties have close contact and interaction with each other, while those with weaker mechanical properties have more wear and deformation. Zirconia is a commonly used material for the superstructure of implants, and its flexural strength is greater than that of titanium 95, 96. Klotz et al. applied a force of 20 N to 200 N on the titanium and zirconia abutments of titanium implants 97, and found that titanium implants with zirconia abutments appear to a greater extent than titanium implants with titanium abutments. Thus, the latter is less resistant to wear and releases more Ti particles. Tawse-Smith A et al. also found that the loading of the zirconia canopy of a single implant can cause wear on the titanium surface and lead to the Ti particles release 98. Similarly, the interaction between pure titanium and titanium alloys with higher hardness also emerged more deformation and wear of pure titanium implants.

Platform switching is defined as a protocol that includes small-diameter restoration components that have been placed on large-diameter implant restoration platforms. The outer edge of the implant abutment interface is away from the outer edge of the implant platform. Alrabeah et al. combined titanium implants with pure titanium, gold alloy, cobalt-chrome alloy, and zirconia abutments, respectively. It is divided into platform matching and platform switching groups 99,100. It is found that the release of metal in all platform switching groups is lower than that of the platform matching group. The release of titanium is found in all experimental groups, and the wear particles are mainly Ti particles. The average amount of Ti particles is the highest in the implant with the platform matching the gold abutment.

It suggested that materials with similar hardness and mechanical properties should be used as much as possible to reduce the wear on the base when selecting implant materials. Platform-switching has a positive effect in reducing the levels of metal release from the implant-abutment.

### 5.3. Clinical Treatment

Grenón et al. found that the diffusion coefficient of Ti particles changes over time. The one-month diffusion coefficient is more significant than three months postoperatively and released more Ti particles 101. Metal particles and ions are diluted and removed by rinsing the surgical site, reducing the total amount of metal 102. Early release of Ti particles can be effectively reduced by checking the integrity of the cutting tool before use, fully controlling the number of disinfection procedures, and replacing the worn drill bit. Surgery contamination such as suction and swallowing, is also responsible for the distribution of Ti particles in distal organs 11.

Clinicians should pay attention to the effect of instruments used and chemicals on the implant surface to reduce the release of Ti particles during the maintenance. Cha et al. compared the effects of five kinds of mechanical equipment (metal clean tooth tip, thermoplastic clean tooth tip, round brush titanium, titanium bristle brush, and glycine abrasive) on the surface roughness of implants 103. They found that the metal teeth cleaning device has apparent damage to the implant surface. Glycine abrasive seems to grow on the surface of the body surface damage to the minimum, thus releasing the least Ti particles.

Therefore, it suggested that the device with little influence on the surface roughness of implant should be chosen when maintaining the implant.

## 6. Reduction in toxicity

Ti particles can inhibit a variety of cell activities, and promote osteoclast differentiation. Biochemical and immunohistochemical analysis indicates that IL-6, IL-1, and TNF-α and other cytokines are highly expressed in peri-implant tissues and can stimulate bone resorption. Therefore, it is critical to reduce or inhibit the toxicity of Ti particles. Several studies have reported that the release is relate to some open pathways and various active substances, effectively reducing the toxicity (Table 3).

### 6.1. Inhibition of effect of inflammatory cytokines

The release of Ti particles can induce an inflammatory response and promote bone tissue absorption around the implant. Eger et al. found that systematically or locally blocking the release of IL-1β, IL-6, and TNF-α around titanium implants can reduce bone resorption induced by the Ti particles 104. Through the phosphatidylinositol 3-kinase-AKT (Pl3K-AKT) signaling pathway, the production of TNF-α is reduced, which could minimize osteolysis and implant loosening 105. Bacterial endotoxin can also enhance the adverse effects of Ti particles, which reduces the production of TNF-α by PI3K inhibitors on the particles with adherent endotoxin by 70% without increasing cytotoxicity. Similarly, the AKT inhibitor can reduce TNF-α production by 83% without increasing cytotoxicity.

### 6.2. Ceramic coating of bioactive substances

Bioactive substances ceramic coatings on titanium substrates can effectively promote osseointegration and reduce the pro-inflammatory effect of Ti particles on surrounding tissues 106.

Rutile particles are mainly used as enhancers in the manufacture of composite materials 107. Several surface modifications that give rise to an outer ceramic layer of rutile have been developed to improve implant wear and corrosion resistance. The rutile layer enhances the adhesion of osteoblast *in vitro* and improves bone fixation *in vivo* 108-110. Vallés et al. cultured the mononuclear macrophages with Ti particles, and found that the amount of TNF-α and IL-6 released by the cells is higher than that of rutile, and the level of cytokine secretion is lower than Ti particles after cultured with rutile particles 111. Higher biocompatibility of titanium-based implants modified with an outer surface layer of rutile is expected to reduce the toxicity of Ti particles.

Li et al. found that magnesium (Mg) is an anti-inflammatory agent that inhibits inflammation and promotes osteogenesis, so that bone biomaterials have anti-inflammatory effects 112. It inhibits the expression of macrophage M1 markers and pro-inflammatory cytokines, reduces the release of TNF-α after co-cultivation with magnesium ions. Moreover, macrophages grown on Mg-containing ceramic coating surfaces are switched from M1 to M2 phenotype with the stimulation of lipopolysaccharide (LPS), which has the same effect. The integration of Mg in biomaterials can reduce the pro-inflammatory effect of Ti particles on the tissue surrounding the implant.

### 6.3. Using plant extracts

Some plants inhibit titanium particles toxicity by preventing the release of inflammatory cytokines. Quercetin (QUE) is commonly found in plants and exerted anti-inflammatory effects 113, 114. The cytotoxicity of Ti particles is inhibited after pretreatment with QUE. Zhang et al. found that QUE reduced the release of inflammatory cytokines from mononuclear macrophages caused by Ti particles 115. In addition, treatment with QUE can significantly reduce the number of osteoclasts. In the mouse skull osteolysis model, QUE inhibits osteolysis caused by Ti particles *in vivo* by inhibiting the formation of osteoclast.

Luteolin is a highly effective TNF-α, IL-6, and nitric oxide inhibitor. In mouse models of acute and chronic inflammation, oral luteolin inhibits the inflammatory response 116. Shin et al. found that luteolin inhibits osteoclast production and bone resorption caused by macrophages by inhibiting the release of inflammatory cytokines induced by Ti particles 117.

Astragaloside IV (As-IV) is a natural plant extract that increases the activity of osteoblasts and has the potential to treat osteoclast-related diseases, including osteoporosis, periodontal disease, and rheumatoid joints inflammation and loosening of the sterile prosthesis. Li et al. found that intravenous injection of As-IV reduces the osteolysis of mice induced by Ti particles 118.

Above all, it is not difficult to find that controlling or inhibiting the release of inflammatory factors and bone resorption are the main approach to reduce the toxicity of Ti particles. The basic mechanism is to limit inflammation, either by coating of bioactive substances or by using plant extracts. This is closely related to peri-implantitis, which is one of the main causes of implant failure. Therefore, it is necessary for us to pay attention to and study how to reduce the toxicity of Ti particles.

## Conclusion and perspectives

Titanium metal is considered the safest material for implants due to its excellent mechanical properties and biocompatibility. It has been widely used in oral, orthopedics, and plastic surgery. The titanium oxide layer on the implant surface can be damaged by mechanical wear and chemical corrosion in long-term use and daily care, causing the release. It varies in size, shape, and content in local and remote, destroy the bone homeostasis around the implant and further aggravate the inflammatory response of surrounding tissues, which triggers peri-implantitis. The potential impact on other cells, tissues, and organs still needs to be explored. This paper has emphasized the methods of reducing the release and toxicity of Ti particles. However, it is still impossible to eliminate Ti particles and achieve zero release. Currently, 1000 tons of titanium is implanted into patients in various forms every year, but the release of Ti particles has not attracted the attention of clinicians. With the advent of new materials and advances in technology, such as atomic layer deposition technology and so on, it is believed that more methods to prevent and reduce the release of Ti particles will soon be applied to basic and clinical research.

In recent years, the rapid development of nanomedicine has promoted the cross integration of many fields. Various metal nanoparticles including precious metal nanoparticles, transition metal nanoparticles, have been successively used in the field of biomedicine to effectively treat some major diseases, such as cancer and Alzheimer's disease. Regarding the Ti particles and titanium nanoparticles released by dental implants, most of the research has focused on how to inhibit their inflammatory response and bone resorption, and their comparison with different metal particles is rare. Consider whether the Ti particles released on implants can be converted to beneficial effects by adding drugs or in some way, similar to implants carrying natural nanoparticles, which are released into the surrounding tissues and have an impact.

## Figures and Tables

**Tables 1:** *In vitro* studies related to titanium particles

Author (Year)	*In vitro*	Titanium particles size	Conclusion
William J. Maloney, M.D et al. (1993)	Bovine Synovial Fibroblasts	0.1-10mm	Titanium has no obvious effect on hexosaminidase at any concentration. The morphological response of fibroblasts to titanium includes membrane wrinkling and filopodia expansion.
Senna et al. (2015)	Bovine ribs	10nm-20µm	The shear force during insertion changes the surface of the dental implant. Ti particles are generated at the bone-implant interface, especially around the surface.
Eemeli Jämsen et al. (2019)	Mouse Bone Marrow Macrophages (mBMMs)	0.1-7.5 µm	The different states of macrophages (young and old) are not affected by Ti particles, but macrophage polarization affects the inflammatory response induced by Ti particles.
Wen Wu et al. (2019)	Fibroblasts	1-3 mm	T particles disrupt the autophagy of fibroblasts in the interface membrane, up-regulate the expression of ADAM10, and then promote the release of CX3CL1, and ultimately promote the chemotactic migration and recruitment of monocytes/macrophages.
Ning Song et al. (2019)	Trigeminal Root Ganglion (TRG) Neurons	<5 mm	Ti particles might alter the electrophysiological properties of voltage-gated potassium channels (VGPCs) on TRG neurons.

**Table 2:** The size, distribution, detection methods and conclusions of titanium particles in related studies *in vivo*

Author (Year)	Implant surface	Titanium size	Distribution area	Detection methods	Conclusion
Schliephake et al. (1993)	Machined	Round size(15X30μm)	Lung, liver, kidneys(5months)	SEM, BSE probe, EDS, FASS	The wear produces Ti particles, which are distributed between the bone and the implant. It is also taken up by cells and transferred to remote organs.
Tanaka et al. (2000)	TPS	1.8-3.2μm	Bone surface	LM, SEM, X-ray, TEM, electron diffraction	It is necessary to study the impact of Ti particles on the human body.
Meyer et al. (2006)	Sandblasted, TPS, Machined, Acid-etched	20nm	Crestal	SEM, EDS	The wear of titanium near the plasma sprayed surface is the highest, followed by the acid-etched and smooth grating surface that has been sandblasted.
Flatebo et al. (2011)	Anodized	100-5000nm	Surface of oral mucosa	HR-ODM, SEM, LA-ICP-MS, EDS	The combination of LA-ICP-MS (identifying chemical components) and HR-ODM (providing a histological reference) seems to be an effective method for detecting particles in oral tissues.
Xiuli He et al. (2016)		0.5-40μm	Tissue around implant	SEM-EDX, light microscopy	Confirm that the Ti particles are released into the tissues around the human jaw.
Mattias Pettersson et al. (2017)			Tissue around implant	SEM, ICP-AES	The surface structure of the implant is important for the amount of Ti particles released, while the total area and diameter of the implant are not so important.

TPS: titanium plasma-sprayed; SEM: scanning electron microscopy; BSE: back-scattered electron; EDS: energy dispersive X-ray; FAAS: flameless atomic absorption spectroscopy; LM: Light microscopy; TEM: transmission electron microscopy; LA-ICP-MS: laser ablation inductively coupled plasma mass spectroscopy; ICP-AES: Coupled plasma atomic emission spectroscopy.

**Table 3:** Correlative study of different active substances on the inflammatory response and bone resorption induced by Ti particles

Author (Year)	Active substance	Titanium particles size	Inhibit the effects of Ti particles	Mechanism
Zichuan Ping et al. (2017)	Melatonin	3.32 ± 2.39 µm	Inhibition of bone resorption and expression of inflammatory cytokines	Suppression of NF-κB signaling
Ziguan Zhu et al. (2018)	Aucubin	3-4 µm	Inhibit the apoptosis of Mc3t3-e1 cells and promote osteogenesis	Affecting the BMP2/Smads/RunX2 signaling pathway
Chenhao Pan et al. (2019)	20(S)-protopanaxadiol (PDD)	1-3 µm	Inhibition of osteoclast formation and release of inflammatory cytokines	Inhibition of MAPK and NF- B signaling pathways
Ruize Qu et al. (2019)	Ghrelin	3.32 ± 2.39 mm	Inhibit the inflammatory response; Reduce osteoblasts formation injury and bone resorption	Activation of β-Catenin Signaling Pathway
Chao Yang et al. (2019)	Curcumin		Inhibition of osteoclast maturation and formation stimulated by RANKL has an immunomodulatory effect on macrophage polarization	Activates the Akt/NF B/ NFATc1 pathway
Chao Yang et al. (2019)	Puerarin		Inhibition of bone resorption and production of pro-inflammatory cytokines; Inhibition of osteoclast activation.	Reduced RANKL-stimulated MEK/ERK/NFATc1 signaling cascades
Chao Yang et al. (2019)	Lithium chloride		Increases the release of anti-inflammatory and osteocellular factors.	Induction of macrophage polarization, M2 phenotype
Chao Yang et al. (2020)	Naringin	1-3μm	Inhibits the release of inflammatory factors TNF- and IL-6	Inhibit P38 MAPK pathway
Xiang Wei et al. (2020)	DAPT(N-[N-(3,5-difluorohenacetyl)-l-alanyl]-S-phenylglycine tert-butyl ester, GSI-IX)		Inhibition of osteoclast formation and function; Almost no osteoclasts were formed under high concentration DAPT.	Suppressing the RANKL/Notch2 signaling pathway DAPT
Zhenyu Sun et al. (2020)	Magnoflorine		Inflammatory bone resorption was inhibited *in vivo* and osteoclast formation was inhibited *in vitro*	Suppression of MAPK and NF-kB Signaling
Zhiwei Zhang et al. (2020)	Bortezomib (BTZ), Nanosized and Alumina (Al) particles	<5µm	Reduces apoptosis, inflammation and bone resorption	Reduced NF- B activation of -TRCP and decreased expression of Caspase-3

## Abbreviations

Ti particles: titanium particles
SLA: sandblasting acid etching
LA-ICP-MS: laser ablation inductively coupled plasma mass spectroscopy
BMSC: bone marrow mesenchymal stem cells
ROS: reactive oxygen species
MMP: matrix metallopeptidase
ECM: extracellular matrix
IL: interleukin
TNF-α: tumor necrosis factor-alpha
RANKL: receptor activator of nuclear factor-κB ligand
M: macrophages
DDR: DNA damage response
STi: smooth titanium
TPS: titanium plasma-sprayed
Al-SLA: alumina oxide sandblasted and acid-etched
Zr-SLA: zirconium oxide sandblasted, and acid-etched
Pl3K-AKT: phosphatidylinositol 3-kinase-AKT
Mg: magnesium
LPS: lipopolysaccharide
QUE: quercetin
As-IV: astragaloside IV
mBMMs: mouse bone marrow macrophages
TRG: trigeminal root ganglion
SEM: scanning electron microscopy
BSE: back-scattered electron
EDS: energy dispersive X-ray
FAAS: flameless atomic absorption spectroscopy
LM: light microscopy
TEM: transmission electron microscopy
ICP-AES: coupled plasma atomic emission spectroscopy
